# The effect of the *Verbascum Thapsus* on episiotomy wound healing in nulliparous women: a randomized controlled trial

**DOI:** 10.1186/s12906-021-03339-6

**Published:** 2021-06-08

**Authors:** Sahar Taleb, Maryam Saeedi

**Affiliations:** Saveh University of Medical Sciences, Saveh, Iran

**Keywords:** Episiotomy, Wound, Wound healing, *Verbascum Thapsus*, Mullein

## Abstract

**Background:**

The pain and discomfort caused by episiotomy affect the quality of life of the mothers, so rapid and complete repair of the episiotomy is very important. Due to the effective ingredients of *Verbascum Thapsus*, it has been used since ancient times to treat wounds. Therefore, this study aimed to evaluate the effect of *Verbascum Thapsus* on episiotomy wound healing.

**Methods:**

The study was designed as a randomized, double-blind, controlled clinical trial. Ninety-three primiparous women who were referred to Fatemeh Zahra Hospital in Saveh in 2015 were randomly divided into two groups of intervention (*Verbascum Thapsus*) and control (placebo). Both groups covered the episiotomy wound twice a day for 10 days with 2 cm of prescribed creams. Wound healing was assessed using the REEDA scale before the intervention and on days 1,3 and 10 after the intervention.

**Results:**

Before the intervention, there was no statistically significant difference in terms of demographic characteristics, obstetrics, and REEDA scores between the two groups (*p* < 0.05). The mean scores of REEDA on days 1 and 3 in the intervention group were better than the control group but were not statistically significant. However, on the tenth day after the intervention, the mean scores of REEDA were significantly better in the Verbascum group than the placebo (*p* = 0.01).

**Conclusions:**

According to the results of this study, it seems that *Verbascum Thapsus* is effective in repairing episiotomy wounds. The researchers hope that the results of this study can provide clinical evidence for the use of this herbal medicine in the wound healing process.

**Trial registration:**

This study was registered in the Iranian Registry of Clinical Trials (IRCT) with the code “IRCT201404073106N15” on 02/12/2015.

## Background

Episiotomy is a controlled surgical incision on the perineum and posterior wall of the vagina, which is primarily performed to reduce perineal tears (grade 3 and 4) in the second stage of labor [1, 2]. The use of episiotomy to facilitate childbirth has long been part of the midwifery practice introduced by Dr. Joseph Lee more than a century ago [3]. Although episiotomy should be performed based on the judgment of the cause of labor if there is any indication, nevertheless, in many countries, episiotomy is performed routinely [4]. The prevalence of episiotomy in Iran is reported to be 41.5%, which is much higher than the WHO standard [5].

Perineal muscles play an important role in normal activities and cutting this area causes a lot of pain and discomfort for the mother [6, 7], this issue disrupts physical activities and mental problems in the mother [8]. Slow healing of episiotomy wounds is one of the factors that aggravate the problems associated with it [9] and also has a great impact on health care costs [10].

The search for the most ideal method of wound healing has many challenges. Due to the side effects of synthetic drugs and the emergence of antibiotic-resistant bacteria, experts have reviewed traditional methods of wound healing, using complementary and alternative medicine [10]. traditional plants also Having minimal side effects are more cost-effective; Therefore, they can be a better option in wound healing than synthetic compounds [11], but confirmation of the use of herbs requires clinical evidence [12]. Today, the effect of various herbal treatments such as Chamomile [13], lavender [14], *Aloe Vera* [15] Calendula [16], Pineapple [17], lavender [18] for healing episiotomy wounds has been confirmed [19].

The Verbascum is one of the plants that has been proposed as an effective medicine in the treatment and healing of wounds in traditional medicine [20]. This flower with the scientific name of the Verbascum is the largest class of the Scrophulariaceae family and has about 2500 species [21]. In general, plants of this genus are called Mullein [22]. And more than 20 species grow in different parts of Iran [23].

The phytochemical compounds of the Verbascum have therapeutic effects. The results of qualitative phytochemical screening showed that *Verbascum Thapsus* is a rich source of biologically active compounds that may be useful in the treatment of various diseases [24].

Numerous studies in the laboratory confirmed that the extract of this plant has anti-inflammatory properties [25], antibacterial [26, 27], anti-virus [28], analgesic [29], and sedative properties [30]. Toxic effects of this plant have not been reported in any of the studies [25, 29]. In the research conducted, the Toxic effects of this plant have not been seen [25, 29, 31, 32]. Besides, the traditional use of Mullein as herbal medicine is described in the EMA Pharmaceutical Monograph [33].

The chemical structure of the Verbascum contains substances such as tannins, alkaloids, and saponins, polysaccharides, and flavonoids, each of which can be responsible for wound healing activities [31]. The flavonoids and polysaccharides in this plant cause the proliferation of fibroblasts and connective tissue, which causes a significant effect of the Verbascum in the process of healing incisional and excisional wounds [20].

The researchers also concluded that Verbascoside in this plant has an anesthetic and analgesic effect [25]. The experimental study of Akdemir in vitro in two groups of the Verbascum ointment and control showed that Verbascum has significant activity in wound healing, pain relief, and anti-inflammatory properties [25]. In the study of Taleb et al., The effect of the Verbascum cream on episiotomy pain in 100 nulliparous women was investigated and the results were analyzed on days 1, 3, and 10 after the study. The data obtained from the study showed that the Verbascum has a significant effect on reducing the pain intensity score compared to the placebo group in the studied days [34].

In the laboratory, the safety and security of this plant have been confirmed as complementary medicine [20, 28]. The Verbascum is made in the form of oral capsules, alcoholic extracts, oils, creams, and ointments in some countries [21, 25]. The species of this plant, especially *Verbascum Thapsus*, has been known as an herbal medicine in many countries of the world for centuries, and the value of this plant as a medicinal plant has been proven [22]. According to studies, many researchers have stated that in the future, the Verbascum can be used as a useful and cost-effective drug in medicine [28, 35]. Accordingly, the present study aimed to investigate the effect of the *Verbascum Thapsus* on the episiotomy wound healing process.

## Method

### Trial design

This study was designed as a randomized, double-blind, placebo-controlled clinical trial with two parallel arms. This study was reviewed and approved by the ethics committee of Shahid Beheshti University of Medical Sciences with the license number “IR. SBMU2.REC.1394.78” and also was registered in the Iranian Registry of Clinical Trials (IRCT) with the code “IRCT201404073106N15” on 02/12/2015. the full trial protocol can be accessed at: https://en.irct.ir/trial/3984. In this study, The effect of *Verbascum Thapsus* on episiotomy wound healing was compared with placebo.

### Study participants

This study was performed on 100 eligible primiparous women in Fatemeh Zahra Specialized Hospital in Saveh from November to January 2015.

Inclusion criteria included: primiparous mother in the age range of 18 to 35 years who had a vaginal delivery with medial-lateral episiotomy, delivery of a single, term infant with a normal weight range, no rupture more than 3–4 degrees, no history or a current illness that interferes with wound healing and does not take any medications that affect wound healing during the study. Exclusion criteria included: non-use of creams as prescribed, allergy to the Verbascum cream, neonatal hospitalization during the study, need for perineal repair after episiotomy, fever, and puerperal infection.

### Selective process

At the beginning of the study, individuals who wished to participate in the study underwent an initial evaluation, which included demographic and obstetric characteristics such as episiotomy, length, and the number of sutures, and the absence of other perineal ruptures. Having inclusion criteria as a sample were included in the study. All samples were similar in terms of episiotomy type, repair method, type of suture, and amount of anesthetic before cutting and repairing. One hundred volunteer participants were selected for the study after obtaining a written consent; the purpose and method of the study were explained to them. Both groups received face-to-face training on how to use the cream. In this study, by performing the same procedure according to the standards, controlled and sterile conditions were created to prevent episiotomy infection during the procedure.

### Sample size

According to the previous study [36], using the statistical formula of mean difference, considering α = 0.05, β = 0.1, effect size 0.70, and test power 0.80, 45 samples in each group were determined. With a 10% calculation for the probable sample loss, the appropriate number of samples in each group was considered to be 50 people.

### Randomization

The random allocation of samples was such that with the help of Excel software (Random Allocation Software; block size = 2), the numbers 1 to 100 (in the number of samples) were randomly given code 1 or 2 and the code was set 1, ointment A should be delivered and code 2 ointment B should be delivered. Thus, 50 people were in the group of flowers (samples with code 1, ointment A) and 50 people in the placebo group (samples with code 2, ointment B).

The random allocation of samples was such that with the help of Random Allocation Software, the numbers 1 to 100 (in the number of samples) were randomly assigned to code 1 or 2 and it was decided to Code 1 should be delivered with ointment A and code 2 should be delivered with ointment B. Thus, 50 people were in the Verbascum group (samples with code 1, ointment A) and 50 people in the placebo group (samples with code 2, ointment B).

For blinding, samples and researchers involved in therapeutic interventions and study evaluation remained blind until the data analysis was completed. Random allocation and data analysis were also performed by a researcher who was not involved in the study.

### Study medication and intervention

The Verbascum was collected by a botanist from the rangelands of Qazvin province. The plant sample is kept in the herbarium of Jahad Daneshgahi Medicinal Plants Research Institute with the code 4535. The flowers of the plant were first cleaned, washed, and dried in the shade, then pulverized by an electric mill and the extract was prepared by 96% ethanol by maceration method (3 times). Finally, after evaporation of the solvent, pure extract with 16% efficiency was obtained. This extract is mixed with a suitable amount of cream base (Eucerine) prepared from Sepidaj company and packed in 30-g containers. Each can of cream contained 7.5% of plant extract. The safety of this dose was obtained according to previous findings and studies [33]. The same base cream with green color was used to make the placebo. Both the drug and the placebo were coded by the pharmacist (A, B). Thus, the researcher and the samples did not know the contents of the packages. After washing and cleaning the episiotomy area, mothers were asked to apply 2 cm of cream to the episiotomy site twice a day for 10 days and use a clean pad 1 to 2 min later. Both groups received the necessary training on how to use the cream, face to face, by the researcher.

### Assessment

REEDA scale was used to evaluate the healing of episiotomy wounds. This scale was designed by Davidson in 1974 and can be used to examine redness, edema, bruising, discharge, and wound edge adhesion [37]. On the REEDA scale, a score between 0 and 3 is considered for each variable, which is the total score of the variables between 0 (maximum improvement score) to 15 (minimum improvement score). The validity and reliability of this scale have been confirmed by content validity and inter-rater reliability (r = 0.79), respectively [38]. To collect information, demographic information forms, health status and obstetric characteristics, analgesic registration form, antibiotics, and drug side effects were designed. To confirm the validity of these forms, the content and face validity method and the reliability of these forms were confirmed using the test-retest method with a correlation coefficient of 0.88.

### Follow-up, outcomes

Preliminary outcomes included evaluation and comparison of the mean scores of episiotomy wound healing in the group of Verbascum and placebo. The perineal ulcer was evaluated by the researcher using the REEDA scale, before the intervention in the first hours after delivery in the hospital and then during the 1st, 3rd and 10th days after delivery in the hospital clinic in two groups and compared. The drug registration form and the use of antibiotics and analgesics were completed on the mentioned days and in case of any side effects such as irritation, itching, fever and chills, and infection, the drug was immediately discontinued and the sample was referred to a gynecologist. Also, the researcher’s telephone number was provided for the samples to answer their questions. All procedures were performed under the supervision of a gynecologist.

### Measurement of phenolic and flavonoid compounds

The phenolic component of the extract was measured based on gallic acid equivalent by employing the Foline Ciocalteu colorimetry method. Various concentrations of gallic acid (20, 40, 60, 80, 100 g/mL as standard) were prepared in ethanol. Then, from each sample, 1 mL was transferred to a suitable test tube and the Foline Ciocalteu 10% (5 mL) was added to the test tube as a reactive reagent. After 10 min, 4 mL sodium carbonate (7.5 mg/mL) was added to the solution. Then the test tubes were left at room temperature for 30 min and assayed in three intervals. To measure the total phenolic compounds, 10 mg of the prepared extract was dissolved in 10 mL ethanol, and total phenolic content was measured (mg/g extract in gallic acid equivalent) by a spectrophotometer (Unico UV2010, Japan) at 765 nm wavelength.

Total flavonoids of the extract samples were measured equivalent to Rutin as a standard, using the aluminum chloride colorimetry method. For this, various concentrations of Rutin (20, 40, 60, 80, 100 μg/mL) were produced in ethanol. Afterward, 1 mL of each standard sample was added to a suitable tube and mixed with 2.5 mL of 20 mg/mL aluminum chloride sample. Then, the level of optical density was read 40 min later at 415 nm wavelength. The total concentration of flavonoids in the extract was measured the same as the method for phenolic compounds, but equivalent to Rutin.

### Statistical analysis

Data were analyzed using SPSS software version 16. Descriptive statistics were used to describe the data in the groups, including central indicators and dispersion and frequency distribution. The Chi-square test was used to compare the groups in terms of qualitative variables and the independent t-test and Mann-Whitney nonparametric test was used to compare the groups in terms of quantitative and rank variables, respectively. In all tests, a significance level of 0.05 and a Confidence Interval of 95% were considered.

## Results

### The main results

**Fig. 1:**
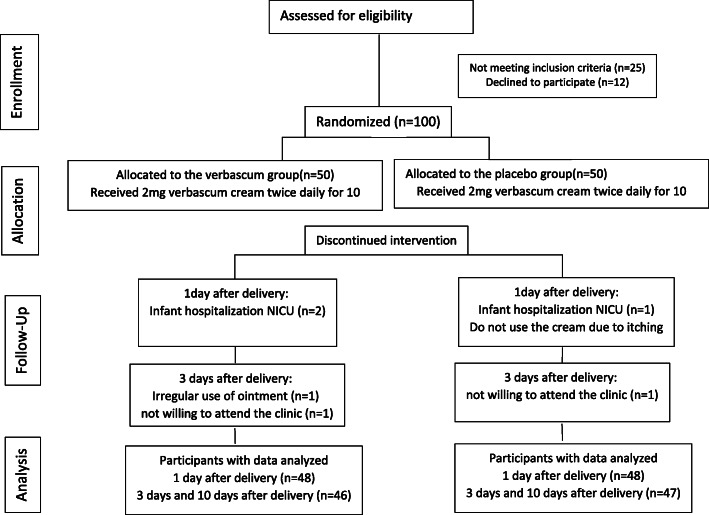
Flow chart of the participants through each stage of the trial

During the study, out of 100 samples that met the inclusion criteria, seven samples were excluded due to the exclusion criteria. In the first 3 days after the intervention, four samples left the intervention group and three samples left the control group. Finally, the analysis was performed on 93 nulliparous women (46 in the Verbascum group and 47 in the placebo group). The number of samples involved in the research is shown in Fig. 1.

Most of the samples in the intervention group (26 samples, 56.5%) and in the control group (24 samples, 51.1%) had primary and secondary education, which according to the Mann-Whitney test, no significant difference was observed between the two groups (*P* = 0.7).

Thirty-six samples (78.3%) from both groups were housewives. Most of the samples had a good income in the intervention group (39.1%) and moderate-income in the control group (40.4%). The results of the Mann-Whitney test did not show a statistically significant difference between the two groups in terms of income level (*P* = 0.65). The health status of most samples was good; eighteen samples (39.1%) in the Verbascum group and 22 samples (46.8%) in the placebo group were in good health. Also, the Mann-Whitney test did not show a statistically significant difference between the two groups in this regard (*p* = 0.48). The duration of the first, second, and third stages of labor was assessed by independent t-test and no significant difference was observed between the two groups (*P* > 0.05). There was no statistically significant difference between the two groups in terms of other demographic characteristics and delivery information (P > 0.05) (Table 1). There was no statistically significant difference between the two groups in the mean scores of REEDA before the intervention. Although the mean score of REEDA in the Verbascum group was lower than the control group in the first and third days after the intervention, the Mann-Whitney test did not show a statistically significant difference between the two groups. On the tenth day after the intervention, the majority of the samples in the Verbascum group did not have any redness (73.9%), edema (91.3%), discharge (89.1%), and wound opening (78.3%) at the episiotomy site and the difference in mean REEDA score was significant using Mann-Whitney test in both groups (*p* < 0.001) (Tables 2 and 3). No bruising was seen at the episiotomy site in any of the research units.

**Table 1:** Comparison of the frequency distribution of research units in terms of demographic characteristics and midwifery information in the intervention and control groups

Demographic and obstetric information	Placebo group (*n* = 47)	Verbascum group (*n* = 46)	*p*-value
Mother age (years)	24.17 ± 3.66	22.91 ± 3.62	^a^*P* = 0.516
Body mass index (BMI)	23.64 ± 3.47	23.35 ± 3.44	^a^*P* = 0.682
Gestational age (weeks)	38.47 ± 1.03	38.54 ± 1.1	^b^*P* = 0.776
Baby weight (grams)	3227.66 ± 326	3216.30 ± 344	^a^*P* = 0.543
Duration of episiotomy repair	18.26 ± 7.94	18.33 ± 8.64	^a^*P* = 0.978
Episiotomy length	4.62 ± 1.27	4.54 ± 1.37	^a^*P* = 0.774
Number of episiotomy sutures	4.67 ± 1.33	4.70 ± 1.42	^a^*P* = 0.994
Number of threads used for episiotomy sutures	2.88 ± 0.75	3.29 ± 0.74	^b^*P* = 0.599

Mean ± Standard deviation for quantitative variables and percentage for qualitative variables

^a^ Independent T-test

^b^ Mann-Whitney U test

**Table 2:** Comparison of central and dispersion indices of episiotomy wound healing scores in the first, third, and tenth days after intervention in intervention and control groups

Group	Descriptive statistics	One day after intervention	Three days after the intervention	Ten days after the intervention
Verbascum	Mean ± Std. Deviation	4.70 ± 2.882	3.26 ± 2.498	.67 ± 1.283
	Median	4.00	3.00	.00
	Minimum	0	0	0
	Maximum	11	10	6
	N	46	46	46
Placebo	Mean ± Std. Deviation	4.94 ± 2.777	3.87 ± 2.401	2.26 ± 1.847
	Median	4.00	3.00	2.00
	Minimum	1	0	0
	Maximum	11	10	9
	N	47	47	47
*P*-Value^a^		0.68	0.23	0.000

^a^ Mann-Whitney U test

**Table 3:** Comparison of the frequency of patients improved in the first, third, and tenth days after the intervention in the intervention and control groups

Group	Total Number	One day after intervention	Three days after the intervention	Ten days after the intervention
Verbascum^a^	46	1 (2.17%)	5 (10.86%)	32 (69.56%)
Placebo^a^	47	0 (0%)	1 (2.13%)	7 (14.89%)
*P*-Value^b^	0.000			

^a^ Values are expressed as frequency (percent)

^b^ Chi-square test

### Extract analysis

The total content of phenolic compounds in the Verbascum was 60.13 μg/ mL gallic acid equivalent and the total content of flavonoids was 20.64 μg/ mL Rutin equivalent in dry extract.

### Safety

In the analysis of the frequency distribution of samples for side effects, no skin itching (pruritus) was observed in the intervention group. In the placebo group, one sample (2.1%) reported itching on the first day of the intervention. The Chi-square test did not show a statistically significant difference between the two groups (*p* = 0.912). No cases of infection, irritation, and dryness at the episiotomy site were reported and observed in the samples until 10 days after the intervention.

## Discussion

In the present study, we investigated the effect of the *Verbascum Thapsus* cream on the episiotomy section. The results of data analysis of the mean REEDA score showed that Verbascum had a better effect on episiotomy healing compared to the control group, which was significantly higher than the control group on the tenth day of the intervention. In a review of the literature, no similar study was found on the effect of the *Verbascum Thapsus* cream on episiotomy wounds, and this is the first study to investigate this issue.

The effective and diverse compounds in the Verbascum plant species such as flavonoids, polysaccharides, tannins, saponins, verbacosides, steroids, carbohydrates, and anti-inflammatory and antimicrobial properties are important factors in the regeneration of dermis [20, 25, 31, 32]. As the results of the extraction of the Verbascum chemical compounds in our study showed, one of the most abundant compounds of this plant is the flavonoid, which is consistent with the results of other studies [21, 39]. Flavonoid wound healing mechanisms include increased vascular endothelial growth factor (VEGF), growth factor and hydroxyproline, and anti-inflammatory effects [40].

In the present study, another compound extracted from the *Verbascum Thapsus* was phenol. Plant extracts that have high phenolic components, due to their antioxidant, antibacterial, and anti-inflammatory activity, have a high potential for rapid repair of damaged skin tissues; Besides, through stimulating collagen synthesis by fibroblasts, they can help increase wound consistency [41]. In the study of Yadav (2018), the use of phenol-rich topical ointment potentially accelerated the hydroxyproline content and collagen synthesis in the wound [42]. According to the results of these studies, phenol and flavonoid compounds extracted from Verbascum can be effective factors in wound healing.

According to phenological studies, Verbascum flowers bloom from June to September and this is the time of harvest of this plant [43]. A review of studies shows that the amount of chemical compounds such as phenol and flavonoids in the plant depends more on the type of plant organ, the type of species, and the place of plant growth and environmental factors than on the time of harvest [43–45]. The results of the studies showed that the extract of *V. sinuatum* contains the highest amount of phenolic compounds and antioxidant activity [44, 46, 47]. In the study of Selseleh et al., The contents of phenol and flavonoids of *V. sinuatum* were higher than that of *V. Thapsus* [47]. Moreover in the study of Karimian and Ghasemlou, the phenolic compounds of *V. sinuatum* (118.2 ± 2.46 mg / g dry weight) were higher than the phenols of *V. Thapsus* in the present study. As well as, the flavonoid content of *V. speciosum* (5.77 ± 0.23 mg QE / g DW) was higher than our study species [46]. Therefore, it is suggested that further studies be performed to compare different species of Verbascum on episiotomy wounds.

Although no similar study of the effect of the Verbascum on episiotomy wound has been seen in the literature, many medicinal plants have a similar composition to this plant and their effects on episiotomy wound healing have been investigated. One of the plants that have compounds and properties similar to the Verbascum is horsetail or *Equisetum arvense* [48]. The results of Asgharikhatooni et al.’s study showed that *Equisetum arvense* ointment caused a significant improvement in episiotomy wounds in nulliparous women [49]. In the study of Shahrahmani et al., The effect of green tea ointment on pain and healing of episiotomy wounds was investigated. In this study, phenol and flavonoids are the extracted compounds of this plant and the comparison of REEDA scores showed healing and acceleration of episiotomy wounds in the intervention group [50], which is per the present findings.

Due to its anti-inflammatory power and stimulant effects of wound healing, the Verbascum was a promising therapeutic approach in wound healing. In the study of Sohrabi-Haghdost et al. [51] the effect of the *Verbascum Thapsus* extract on wounds in rabbits was compared with zinc oxide; The results of histopathological observation of the wound site in the Verbascum group showed young granular tissue rich in fibrin, inflammatory cells, and young epithelial tissue with angiogenesis. Angiogenesis is one of the basic processes of wound healing [52]. The Verbascum has angiogenesis and expression of genes associated with wound healing effects [20].

In Nebiuni et al.’s study, the effect of the smoke from burning Verbascum leaves on Wistar rat wound healing was investigated and improvement in wound healing of rat was observed in the Verbascum plant group compared to the control group. Factors such as increasing the thickness of the epidermis, angiogenesis to accelerate the process of blood flow to the wound site, along factor expression (VEGF) have been mentioned as wound healing mechanisms of this plant [53].

In the proliferative stage of wound healing, due to the migration of myofibroblast cells, the edges of the wound become closer together and the wound size decreases. This process lasts from the second to the fourteenth day of wound healing [54]. The elements in the Verbascum cause the proliferation and migration of fibroblast cells to the wound site [20]. In the present study, better closure and healing of the episiotomy site in the Verbascum group confirms the effect of this plant on wound healing.

Another study in India, entitled The effect of the *Verbascum Thapsus* on normal and dexamethasone-suppressed wounds in Albino rats, showed that the phytochemical compounds in this plant significantly increase the rate of wound excision closure by increasing the epithelialization process, increasing the volume of granular tissue, and the hydroxyproline content [31]. Considering that collagen is the main component of connective tissue scar in wound healing [54] and according to the results of studies that have shown a significant effect of the Verbascum extract in the collagen formation phase, one of the reasons for wound healing can be attributed to this issue [31].

The *Verbascum Thapsus* reduces swelling and inflammation of the wound site [51]. It seems that the improvement of redness and secretions at the episiotomy site in this study can be attributed to the anti-inflammatory and antioxidant effects of the compounds of this plant [20, 51, 55].

In the present study, better healing of episiotomy wounds was seen in the Verbascum group compared to the placebo group, and the possible mechanisms and effects of phenol and flavonoid compounds of this plant in the wound healing process were discussed. It is hoped that further clinical trials will provide the necessary evidence for the mass and safe use of this herbal medicine. One of the limitations of this study is the inability to control all factors affecting wound healing, such as differences in the individual immune system; However, in this study, we tried to control the intervening factors to some extent by providing similar training to individuals, random allocation of samples and considering inclusion criteria. Study power includes the assessment of episiotomy wound repair with REEDA scale, control of confounding variables, training and evaluating by the same researcher for each group, episiotomy incision, and suture by a trained midwife and according to standard protocol, using the same suture thread and the same amount of anesthesia for all samples. No dissatisfaction was reported from the samples in the two groups during the study.

## Conclusion

According to the results of this study, it seems that *Verbascum Thapsus* cream is effective in repairing episiotomy wounds. The researchers hope that the results of this study can provide the necessary clinical evidence for the use of this herbal medicine in the wound healing process. However, more clinical studies are needed to evaluate the safety and efficacy of the Verbascum before accepting treatment as an evidence-based therapy.

## Data Availability

The datasets used to support the findings of this study are available from the corresponding author on reasonable request.

## Abbreviations

REEDA: Redness, Oedema, Ecchymosis, Discharge, Approximation
CONSORT: Consolidated Standards of Reporting Trial
